# Assessing breast cancer cell lines as tumour models by comparison of mRNA expression profiles

**DOI:** 10.1186/s13058-015-0613-0

**Published:** 2015-08-20

**Authors:** Krista Marie Vincent, Scott D. Findlay, Lynne Marie Postovit

**Affiliations:** Department of Oncology, Faculty of Medicine and Dentistry, University of Alberta, 114th St and 87th Avenue, Edmonton, AB T6G 2E1 Canada; Department of Anatomy and Cell Biology, Faculty of Medicine and Dentistry, University of Western Ontario, 1151 Richmond Street, London, ON N6A 3K7 Canada

## Abstract

**Introduction:**

Breast cancer researchers use cell lines to model myriad phenomena ranging from DNA repair to cancer stem cell phenotypes. Though appropriate, and even requisite, for many studies, the suitability of cell lines as tumour models has come into question owing to possibilities of tissue culture artefacts and clonal selection. These issues are compounded by the inability of cancer cells grown in isolation to fully model the in situ tumour environment, which also contains a plethora of non-tumour cell types. It is thus important to understand similarities and differences between cancer cell lines and the tumours that they represent so that the optimal tumour models can be chosen to answer specific research questions.

**Methods:**

In the present study, we compared the RNA-sequencing transcriptomes of a collection of breast cancer cell lines to transcriptomes obtained from hundreds of tumours using The Cancer Genome Atlas. Tumour purity was accounted for by analysis of stromal and immune scores using the ESTIMATE algorithm so that differences likely resulting from non-tumour cells could be accounted for.

**Results:**

We found the transcriptional characteristics of breast cancer cell lines to mirror those of the tumours. We identified basal and luminal cell lines that are most transcriptionally similar to their respective breast tumours. Our comparison of expression profiles revealed pronounced differences between breast cancer cell lines and tumours, which could largely be attributed to the absence of stromal and immune components in cell culture. A focus on the Wnt pathway revealed the transcriptional downregulation or absence of several secreted Wnt antagonists in culture. Gene set enrichment analysis suggests that cancer cell lines have enhanced proliferation and glycolysis independent of stromal and immune contributions compared with breast cancer cells in situ.

**Conclusions:**

This study demonstrates that many of the differences between breast cancer cell lines and tumours are due to the absence of stromal and immune components in vitro. Hence, extra precautions should be taken when modelling extracellular proteins in vitro. The specific differences discovered emphasize the importance of choosing an appropriate model for each research question.

**Electronic supplementary material:**

The online version of this article (doi:10.1186/s13058-015-0613-0) contains supplementary material, which is available to authorized users.

## Introduction

Since the establishment of the HeLa cell line in 1951, cell lines have been an integral part of cancer research, and their use has tremendously advanced understanding of molecular cancer biology [1]. However, the suitability of these models has come into question, as many in vitro phenomena are challenging to replicate in vivo. Interpreting the potential clinical significance of discoveries made using cell lines requires an understanding of the extent to which these cell lines represent in vivo tumours.

Since the first breast cancer cell line, BT-20, was established in 1958 [2], various other immortalized primary tumour cell lines have been established at exceptionally poor efficiencies [3, 4]. This low efficiency has often been attributed to slow growth rates of tumour cells in culture as compared with associated stromal cells, such as fibroblasts [5]. To overcome this issue, most established breast cancer lines have been derived from pleural effusions, which provide an abundance of dissociated, aggressive tumour cells with very few contaminating cell types. The pattern of growth of these tumour cells is characterized by a slow initial proliferation, followed by exponential expansion of a few cells, suggestive of clonal selection for cells that are particularly proliferative and amenable to culture [6–8].

Another caveat of cell culture is the loss of the in vivo microenvironment (changes summarized in [9]). During the derivation process, tumour cells are removed from a very complex, partially hypoxic three-dimensional microenvironment; maintained in nutrient media supplemented with a surplus of growth factors, including glucose; and passaged indefinitely at relatively high atmospheric oxygen levels. In such a drastically altered microenvironment, it would not be surprising if cell lines differed substantially from the tumours they were established to represent.

Genomic and transcriptional differences between cancer cell lines and tumour samples have been investigated in several studies [10–13]. For example, in gliomas, it was shown that expression profiles of tumour cell primary cultures were much closer to profiles obtained from clinically resected tumours than to profiles of immortalized cancer cell lines [14]. In breast cancer, clustering based on expression profiles has elucidated the many clinically relevant subtypes in cell lines and tumours (summarized in [15]) [16–20]. However, modern RNA-sequencing (RNA-seq) data have not yet been used to directly compare the expression profiles of breast cancer cell lines with breast tumours. As well, in vitro signatures are the combined effect of adaptation to cell culture and selection for specific cellular subtypes. Dissecting out the influence of either of these two phenomena has remained a substantial obstacle in any cell line–tumour transcriptional comparison.

Recent transcriptional profiling of a collection of breast cancer cell lines [21] and hundreds of tumours from The Cancer Genome Atlas (TCGA) [19] has enabled a direct mRNA comparison of cell lines and tumours. In this study, we focus on RNA-seq transcriptional profiles mined from TCGA and the Gene Expression Omnibus (GEO) series [GEO:GSE48213] and investigate the strengths and weaknesses of cell lines as in vitro breast cancer models. In addition, we seek to identify the breast cancer cell lines that are most transcriptionally representative of their respective tumour subtype. Importantly, we are able to correlate most of the highly differentially expressed genes to tumour stromal or immune signatures, highlighting the importance of considering the entire niche in cancer modelling. Finally, we summarize relevant breast cancer cell line genomic alterations. In our study, we used RNA-seq data to broaden the dynamic range of transcript detection and extend earlier efforts by including more cell lines and by considering and quantifying stromal and immune cell contributions to help elucidate the origin of detected differences.

## Methods

### Datasets

Level 3 TCGA RNAseqV2 gene expression data were obtained from the TCGA Data Portal [22] in August 2014. RNA-seq expression data were retrieved in September 2014 for 50 luminal and basal breast cancer cell lines profiled in the GEO database [GEO:GSE48213] [21]. Oestrogen receptor (ER) status and subtype data were available in the original publication for cell lines and were accessed via the UCSC Cancer Genomics Browser (RNASeqV2 defined [23]) for tumours in September 2014. Breast cancer cell line copy number information and mutation data for 1651 genes were retrieved from the cBio Cancer Genomics Portal [24] for the Cancer Cell Line Encyclopaedia (CCLE) in March 2015.

### Data preparation

Relative abundance (in transcripts per million [TPM]) was calculated for 975 breast tumours by multiplying the scaled estimate data by 10^6^, and for 50 breast cell lines by converting fragments per kilobase of exons per million mapped reads to TPM. To avoid infinite values in log calculations, a value of 1 was added to all TPM values before log_2_ transformation. Values for the genes that were available in both datasets (16,282 coding genes in total) were combined for further analysis.

### Gene expression profiling analysis

The top 5000 genes by variance across the combined dataset were chosen for principal component analysis as well as for hierarchical clustering using 1 − *c* (where *c* is Pearson’s correlation coefficient) as the distance and Ward’s agglomeration method (ward.D2). The 5000 most variable genes were also used to compute the Pearson’s correlation coefficient of all the cell line–tumour pairs in a subtype-specific manner. The cell lines were ranked based on their average correlation with all tumours of their respective subtype. Significant differences in relative transcript abundances between cell lines and tumours were calculated with Welch’s *t* test, and *p* values were corrected for multiple testing using the Benjamini-Hochberg method. Enrichment for functionally related genes between the two datasets was tested using Generally Applicable Gene-set Enrichment (GAGE v2.12.3; Bioconductor: [25]) with Kyoto Encyclopedia of Genes and Genomes [26] gene sets with fewer than 200 items.

### Tumour purity

Stromal and immune scores were defined for tumours by ESTIMATE scores (Estimation of STromal and Immune cells in MAlignant Tumour tissues using Expression data) using RNASeqV2 data as previously described [27], and accessed in October 2014 via the bioinformatics portal at the Department of Bioinformatics and Computational Biology, University of Texas MD Anderson Cancer Center [28]. Pearson’s correlation coefficient was used to calculate the association of specific genes with stromal and immune signatures. To decrease hits of transcripts more likely due to tumour purity issues, transcripts that correlated with stromal or immune scores (|*r*|>0.2) were filtered from the list of differentially expressed genes, and a new ranked list was generated. Stromal and immune correlations were calculated for each gene set by averaging the stromal and immune Pearson’s correlation coefficients of the essential genes (as determined using the GAGE package).

### Cell line genomic analysis

The CCLE [29] investigators examined the mutational status of 1651 genes by hybrid capture sequencing and genome-wide copy number analysis. In our genomics summary, we considered all breast cancer cell lines that were available in both the CCLE and at GEO accession number [GEO:GSE48213]. The fraction of the genome altered represents the fraction of the genome that has a log_2_ copy number value above 0.2 or below −0.2. Selected mutational events were considered if they were found to be significantly altered from or in associated healthy tissue in the original TCGA study [19]. Copy number status was investigated for significantly mutated genes in the TCGA study that also displayed frequent copy number amplifications or deletions.

### PubMed citation analysis

The number of PubMed abstracts that mentioned 1 of the 50 breast cancer cell lines was determined as an estimator of frequency of use in laboratories. Hits were determined using the PubMed search function [30] on 18 March 2015. Several punctuation alternatives were used for the cell line names. For the cell lines LY2 and MX1, searches were conducted with the term ‘cells’ to eliminate the inclusion of abstracts that mentioned the *LY2* and *MX1* genes.

### Statistical analysis

We conducted all analyses and visualizations in the RStudio programming environment (v0.98.501; [31]). The R/Bioconductor packages ggplot2, plyr, gplots, ggdendro and GAGE were used as appropriate.

## Results

### Comparison of cell lines and tumour expression profiles

**Fig. 1:**
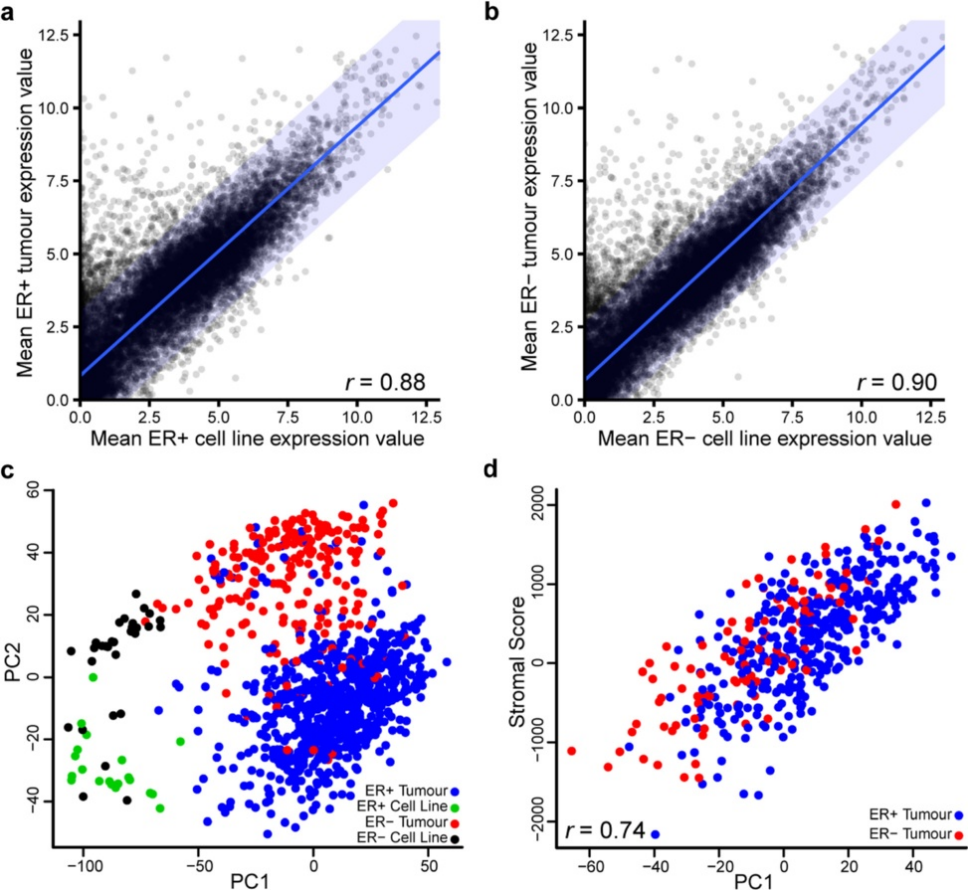
Transcriptional comparison of 50 breast cancer cell lines with 1025 breast cancer tumour samples in The Cancer Genome Atlas suggests overall transcriptional similarity. **a**, **b** Scatterplots of mean expression values (log_2_[transcripts per million + 1]) of 16,282 coding genes in cell lines (horizontal) and tumours (vertical) for oestrogen receptor–positive (ER+) (**a**) and oestrogen receptor–negative (ER−) (**b**) samples reveal that the two datasets are largely comparable with more outliers that are high in tumours and low in cell lines than vice versa. *Blue lines* indicate linear regression; *blue shading* indicates 95 % prediction interval for regression. **c** The 5000 most variable genes were used for principal component analysis, and the first two principal components (PC1 and PC2, respectively) explaining 28 % of the variance are displayed. Cell lines cluster apart from tumours on an axis largely explained by ER status. Colours of the points indicate sample types: ER+ tumour (*blue*), ER− tumour (*red*), ER+ cell line (*green*) and ER− cell line (*black*). **d** PC1 of the breast cancer tumours is highly correlated with ESTIMATE (Estimation of STromal and Immune cells in MAlignant Tumours using Expression data) paradigm stromal scores (*r* = 0.74)

To evaluate the transcriptional fidelity of breast cancer cell lines to tumours, we compared the mean expression values of 16,282 coding genes in oestrogen receptor–positive (ER+) and oestrogen receptor–negative (ER−) cell lines to ER+ and ER− tumours. The mean expression values of cell lines and tumours were similar, though the mean expression values of ER− cell lines and tumours are more highly correlated (*r* = 0.90) than ER+ cell lines and tumours (*r* = 0.88) (Fig. 1a, ER+; Fig. 1b, ER−). However, closer inspection revealed a point of interest: Almost all outliers were genes with high expression in tumours and low expression in cell lines.

We further explored the relationship of cell lines and tumours by conducting principal component analysis (Fig. 1c) and found four clusters clearly divided based on sample group (cell line or tumour; principal component 1 [PC1]) and ER status (principal component 2 [PC2]). One of the main differences between cell lines and tumours is the absence of certain cellular components (e.g., stromal and immune cells). Given that many of the outliers in Fig. 1a, b were genes that had higher expression in tumours than in cell lines, and PC1 in Fig. 1c was largely responsible for the distance between tumours and cell lines, we correlated PC1 with stromal and immune scores in tumours as determined by using the ESTIMATE paradigm [27]. We found that stromal scores strongly positively correlated (*r* = 0.74) with PC1 (Fig. 1d). Thus, the loss of the stromal component likely has significant repercussions in vitro.

In the principal component analysis, we observed that ER− cell lines clustered closer to their respective tumours than ER+ cell lines, indicating again that ER− cell lines may be more representative of their tumour counterparts than ER+ cell lines. Expression-based, unsupervised hierarchal clustering also revealed this trend. Although cell lines cluster apart from all tumours, they cluster closer to the largely ER−/basal subtype division of tumours than to the largely ER+/luminal subtype division (Additional file 1: Figure S1).

### Top differentially expressed genes are genes correlated with stromal and immune scores

**Fig. 2:**
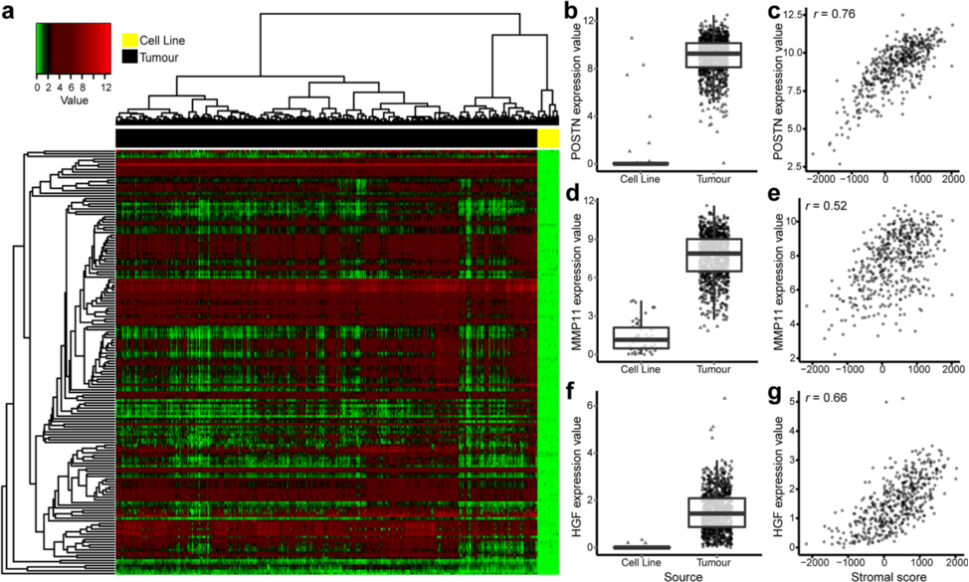
The top 1 % differentially expressed genes in the combined expression dataset. **a** Heatmap representation of the top 1 % of differentially expressed genes (adjusted *p* value by *t* test) in cell lines compared with tumours. In all cases, the direction of difference favoured high expression in tumour samples. All genes were investigated in tumours for potential correlation with the ESTIMATE (Estimation of STromal and Immune cells in MAlignant Tumours using Expression data) paradigm stromal and immune scores. Of the top 163 differentially expressed genes, 134 genes were found to have a Pearson’s correlation coefficient >0.5 (data not shown) with either stromal or immune score. Differential expression (box plot) and stromal correlations (scatterplot) are displayed for representative genes (**b**, **c**) *POSTN*, (**d**, **e**) *MMP11* and (**f**, **g**) *HGF*. Box plots (**b**, **d**, **f**) show gene expression values (log_2_[transcripts per million {TPM} + 1)) stratified by sample source (cell line or tumour). Boxes represent interquartile ranges, and points represent individual sample values. Scatterplots (**c**, **e**, **g**) show gene expression values (log_2_(TPM+1)) versus stromal score (ESTIMATE algorithm) of tumours. Pearson’s correlation coefficient is displayed in the *upper left corner*

We found that the top 1 % of genes differentially expressed in cell culture were all genes that had lower or undetectable expression in culture compared with tumours (Fig. 2). To determine the contribution of stromal and immune cellular compartments to this observation, we correlated the expression of all genes with stromal and immune scores in tumours. We found that 134 of the top 163 differentially expressed genes were highly correlated with stromal or immune scores in tumours (*r* > 0.5). Representative correlations are shown in Fig. 2c, e, g and Additional file 2.

Recently, Winslow et al. investigated different breast cancer cellular compartments using laser capture microdissection [32]. When we examined the genes that they found to be upregulated in breast cancer stromal cells compared with malignant cells, we found that 99 % of them were significantly downregulated in breast cancer cell lines and that their average correlation with the stromal score was 0.65 (data not shown). Taken together, this supports the theory that the downregulation of many genes in cell culture is likely a consequence of losing stromal and immune cellular compartments.

### Wnt antagonists are underrepresented in cell culture

**Fig. 3:**
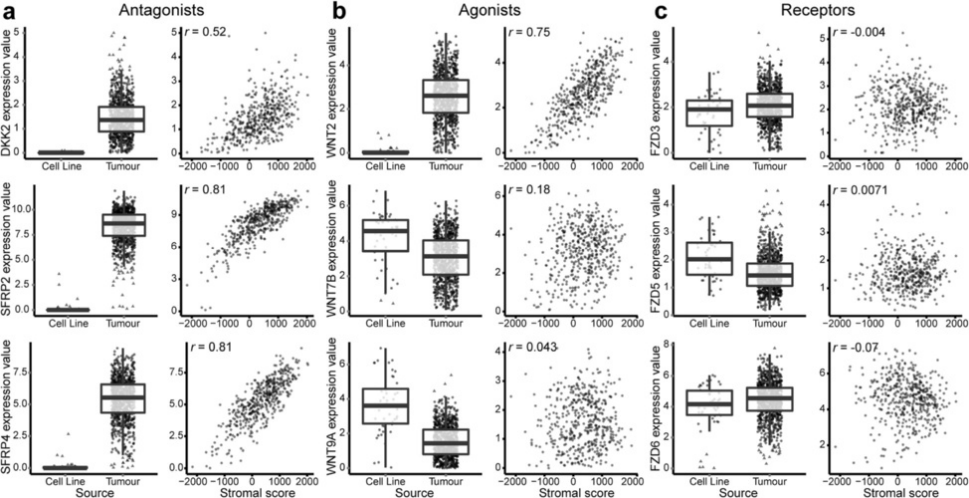
Of the Wnt pathway components, Wnt antagonists (and *WNT2*) are downregulated in cell culture compared with tumours and exhibit a strong correlation with stromal signatures. Differential expression (box plots) and stromal correlations (scatterplots) are displayed for representative (**a**) Wnt antagonists: *DDK2*, *SFRP2* and *SFRP4*; (**b**) Wnt agonists: *WNT2*, *WNT7B* and *WNT9A*; and (**c**) Wnt receptors: *FZD3*, *FZD5* and *FZD6*. Boxes represent interquartile ranges, and points represent individual sample values. Scatterplots (**c**, **e** and **g**) show gene expression values (log_2_[transcripts per million + 1]) versus stromal scores (ESTIMATE algorithm [Estimation of STromal and Immune cells in MAlignant Tumours using Expression data]) of tumours. Pearson’s correlation coefficient is displayed in the *upper left corner*

Because previous studies have suggested that Wnt pathway components are provided by stromal cells in certain situations [33, 34], we investigated the expression of various Wnt pathway members in the datasets. We determined that numerous putative Wnt antagonist transcripts were underrepresented in cell culture and highly correlated with stromal scores (Fig. 3a). However, apart from *WNT2*, all other Wnt agonists (Fig. 3b) and receptors (Fig. 3c) did not display this pattern. This provides evidence that the stromal compartment of tumours provides a unique and non-redundant role in tumours that cannot be modelled accurately using cancer cell monoculture.

### Top differentially expressed genes not correlated with stromal and immune scores

**Fig. 4:**
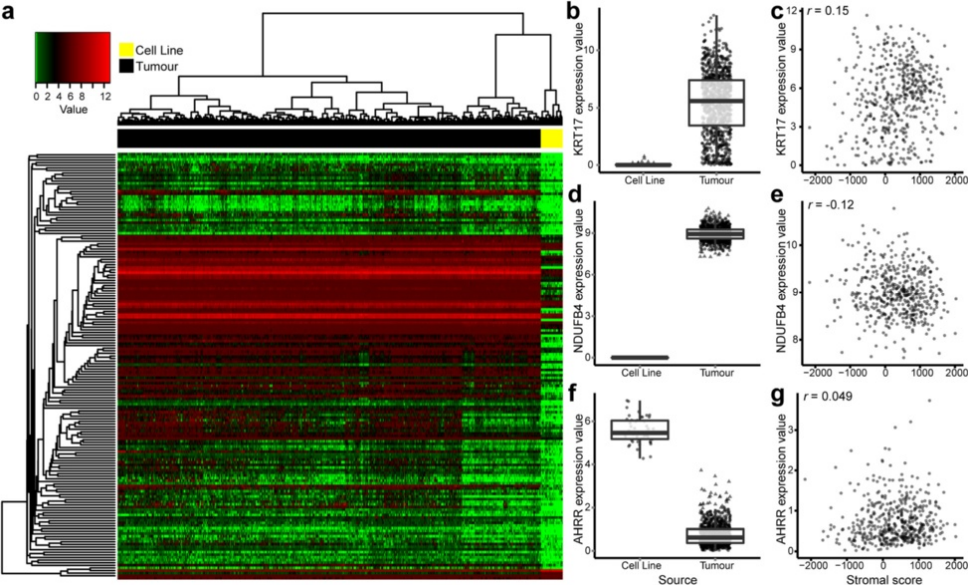
The top 1 % of differentially expressed genes after filtering out genes correlated with stromal and immune scores in tumours. **a** The list of differentially expressed genes was refined for correlation with stromal and immune signatures, uncovering novel differentially expressed genes more likely to genuinely reflect changes induced by cell culture. Heatmap representation of the filtered top 1 % of differentially expressed genes (|*r*| < 0.2; adjusted *p* value by *t* test) in cell lines compared with tumours. Differential expression (box plot) and stromal correlations (scatterplot) are displayed for representative genes: (**b**, **c**) *KRT17*, (**d**, **e**) *NDUFB4* and (**f**, **g**) *AHRR*. **b**, **d**, **f** Boxplots show gene expression values (log_2_([transcripts per million {TPM}+1]) stratified by sample source (cell line or tumour). Boxes represent interquartile ranges, and points represent individual sample values. **c**, **e**, **g** Scatterplots show gene expression values (log_2_[TPM+1]) versus stromal scores (ESTIMATE algorithm [Estimation of STromal and Immune cells in MAlignant Tumours using Expression data]) of tumours. Pearson’s correlation coefficient is displayed in the *upper left corner*

It is expected that cell line in vitro signatures are a combined result of selection for the malignant subtype of cells and in vitro adaptation. Though the expression level changes connected to stromal and immune contributions seem to be the most pronounced, we were interested in investigating subtler distinctions that are more likely a result of in vitro adaptations. In an attempt to overcome the contributions from stromal and immune cell lineages, we identified a new top 1 % of differentially expressed genes after removing the genes whose expression correlated with stromal or immune scores (|*r*| > 0.2) (Fig. 4 and Additional file 3). This list is more likely to reflect changes in cancer cells induced by the cell-culturing process.

**Fig. 5:**
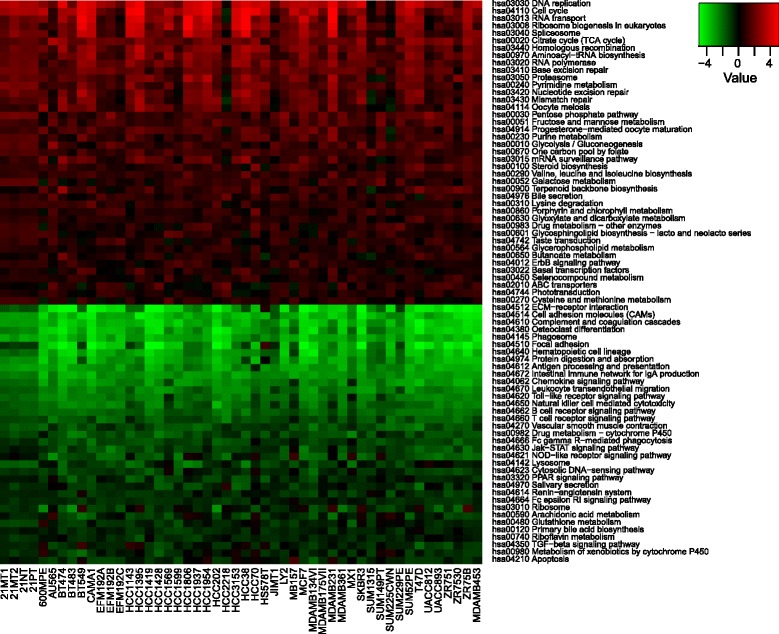
Gene set–specific differences between breast cancer cell lines and tumours. Unpaired Generally Applicable Gene-set Enrichment analysis reveals 41 upregulated and 35 downregulated pathways in breast cancer cell lines compared with tumours with a cutoff false discovery rate *q* value < 0.0001. Heatmap displays generally applicable gene set enrichment test statistics of the 50 cell lines investigated

### Gene set enrichment analysis reveals the enrichment of proliferative gene sets in cell culture

Gene set enrichment analysis revealed 41 upregulated and 35 downregulated gene sets in cell lines compared with tumours (Fig. 5). Cell lines were enriched for gene sets associated with proliferation and metabolism, whereas gene sets associated with extracellular matrix interactions were underrepresented, akin to a pattern that was previously observed when tumours and cell lines were compared [35]. Stromal and immune correlations were calculated for each pathway’s essential genes and averaged for the entire pathway to provide a measure of the contribution of stromal and immune components to resultant gene set perturbation (Table 1). We determined that downregulated gene sets were quite strongly correlated with stromal and immune scores. Specifically, genes that allow tumour cells to interact with extracellular components, such as syndecan 2 and *CD36*, were lower in cell lines than in tumours. However, upregulated gene sets were not as strongly correlated with stromal and immune scores, indicating that their perturbation is likely a result of the differences in the tumour cells themselves.

**Table 1:** Top five upregulated and downregulated KEGG gene sets by gene set enrichment analysis

KEGG gene set	Mean *t* statistic	Set size	Mean correlation to stromal score	Mean correlation to immune score	Mean correlation to tumour purity
Upregulated sets					
hsa03030 DNA replication	3.13	35	−0.27	−0.05	−0.18
hsa04110 Cell cycle	2.85	113	−0.10	0.06	−0.01
hsa03013 RNA transport	2.34	130	−0.13	0.00	−0.07
hsa03008 Ribosome biogenesis in eukaryotes	2.30	61	−0.16	0.02	−0.08
hsa03040 Spliceosome	2.06	103	−0.09	−0.04	−0.07
Downregulated sets					
hsa04512 ECM–receptor interaction	−3.66	81	0.49	0.11	0.33
hsa04514 Cell adhesion molecules	−3.57	120	0.33	0.30	0.36
hsa04610 Complement and coagulation cascades	−3.57	67	0.49	0.34	0.46
hsa04380 Osteoclast differentiation	−3.41	117	0.44	0.49	0.53
hsa04145 Phagosome	−3.35	126	0.37	0.38	0.43

*Abbreviations: KEGG* Kyoto Encyclopedia of Genes and Genomes

GAGE mean *t* statistic and gene set size are reported for the top five up- and downregulated pathways as determined by gene set enrichment analysis. Stromal and immune correlations were calculated for each set’s essential genes and averaged for the entire pathway to provide an estimate of stromal and immune contribution to gene set perturbation

### Ranking of cell lines by transcriptional similarity to their tumour counterparts

**Fig. 6:**
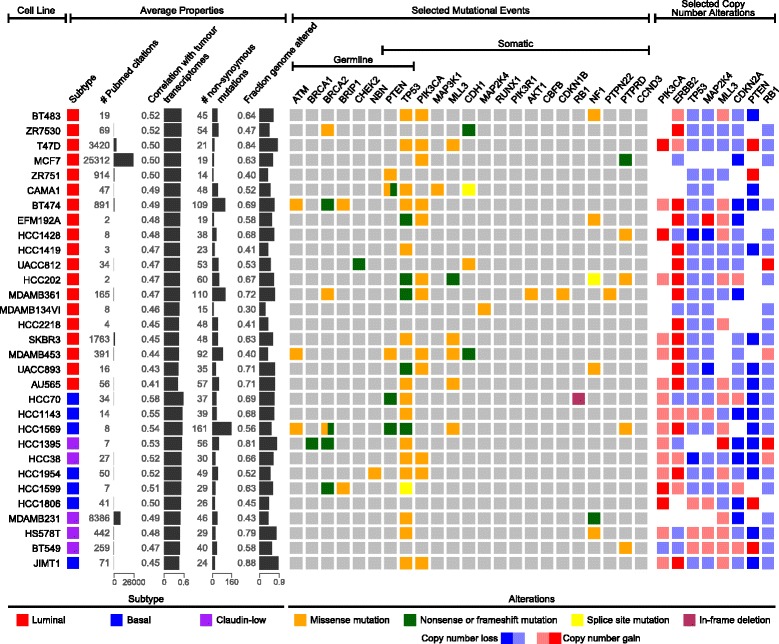
Genomic summary of breast cancer cells lines. Both average properties (*left*) and selected genetic events (*right*) specific to breast cancer can be used to distinguish when to use certain breast cancer cell lines. Average properties include the breast cancer subtype (luminal, basal or claudin-low), the citation frequency in the literature as an estimate of frequency of use, the average transcriptional correlation with tumours of the same subtype as determined in this study, the number of non-synonymous mutations in 1651 genes sequenced by hybrid capture, and the altered fraction of the genome. The selected genetic events include 8 possible germline mutations (*ATM*, *BRCA1/2*, *BRIP1*, *CHEK2*, *NBN*, *PTEN* and *TP53*), 17 possible somatic mutations (*PTEN*, *TP53*, *PIK3CA*, *MAP3K1*, *MLL3*, *CDH1*, *MAP2K14*, *RUNX1*, *PIK3R1*, *AKT1*, *CBFB*, *CDKN1B*, *RB1*, *NF1*, *PTPN22*, *PTPRD* and *CCND3*) and 8 possible copy number alterations (*PIK3CA*, *ERBB2*, *TP53*, *MAP2K4*, *MLL3*, *CDKN2A*, *PTEN* and *RB1*) determined to be significant in the original breast cancer study for The Cancer Genome Atlas and available on Cancer Cell Line Encyclopaedia platforms

To assess the transcriptional suitability of individual cell lines as tumour models, we calculated the correlation coefficients of the top 5000 most variable genes in all subtype-specific cell line–tumour pairs and ranked the cell lines based on their average correlation coefficient (Table 2, Fig. 6). Although this evaluation is not fully comprehensive of all potential genomic and epigenomic differences, it does provide a reasonable guide for choosing cell lines that are most transcriptionally representative of their respective tumour subtype. Ranking the breast cancer cell lines based on correlation leads to a spread of the cell lines from most representative (Table 2, *top*) to least representative (Table 2, *bottom*). Keeping with the trend previously observed, the highest ranked basal cell line (HCC70; *r* = 0.58) was more strongly correlated with respective tumours than the highest ranked luminal cell lines (BT483; *r* = 0.52). It is also reassuring to note that two of the most extensively published luminal cell lines, T47D and MCF7, are ranked fourth and fifth, respectively, of the 27 luminal lines that were evaluated. However, the top ranked luminal and basal cell lines (luminal: BT483, ZR7530 and 600MPE; basal: HCC70, MX1 and HCC3153) are infrequently used as breast cancer models and account for only 0.4 % of publications on this cell line panel.

**Table 2:** Ranking of 50 breast cancer cell lines based on average Pearson’s correlation coefficient of their expression profiles with those of their respective subtype breast cancer tumour samples from The Cancer Genome Atlas

Cell lines	Mean correlation of expression profile to tumours	PubMed citations, *n*
Luminal cell lines		
BT483	0.5204	19
ZR7530	0.5165	69
600MPE	0.5137	20
T47D	0.5028	3420
MCF7	0.5016	25312
ZR751	0.4974	914
CAMA1	0.4892	47
BT474	0.4852	891
EFM192A	0.4837	2
HCC1428	0.4773	8
SUM225CWN	0.4772	11
HCC1419	0.4733	3
UACC812	0.4730	34
HCC202	0.4680	2
MDAMB361	0.4677	165
ZR75B	0.4629	17
EFM192B	0.4612	0
EFM192C	0.4593	0
MDAMB175VII	0.4568	21
MDAMB134V1	0.4566	8
LY2	0.4554	84
HCC2218	0.4505	4
SUM52PE	0.4480	19
SKBR3	0.4456	1763
MDAMB453	0.4447	391
UACC893	0.4320	16
AU565	0.4143	56
Basal cell lines		
HCC70	0.5756	34
MX1	0.5745	23
HCC3153	0.5634	4
HCC1143	0.5502	14
HCC1937	0.5491	145
HCC1569	0.5429	8
HCC1395	0.5276	7
MB157	0.5260	60
SUM149PT	0.5196	13
HCC38	0.5167	27
HCC1954	0.5165	50
SUM229PE	0.5165	6
HCC1599	0.5109	7
HCC1806	0.5042	41
21NT	0.4986	10
21PT	0.4912	28
MDAMB231	0.4871	8386
21MT2	0.4833	8
HS578T	0.4760	442
21MT1	0.4742	10
BT549	0.4700	259
JIMT1	0.4528	71
SUM1315	0.4184	26

The 5000 most variable genes were used to compute the Pearson’s correlation of all the cell line–tumour and cell line–cell line pairs in a subtype-specific manner. The cell lines were ranked based on their average correlation with all tumours of their respective subtype. Claudin-low cell lines were compared with basal tumours, as the claudin-low subtype is not well represented in vivo

We went further and investigated mutation status and copy number alterations of breast cancer cell lines profiled by the CCLE. With our transcriptional correlation ranking, we created a summary of all of these events (Fig. 6), hoping that it could help inform breast cancer cell line choice. The frequency of these somatic mutational events in cell lines mirrors the frequency found in tumours, with a few notable differences: *TP53*, *PTEN*, *NF1* and *PTPRD* were mutated at significantly higher frequencies in cell lines as compared with tumours (*p* < 0.05 by binomial test) (Additional file 4).

## Discussion

This study is the first transcriptional comparison of cancer cell lines and tumours to methodologically account for the contributions of tumour stromal and immune cellular components. We demonstrate, for the first time to our knowledge, using RNA-seq data, that breast cancer cell lines generally represent breast tumours, with notable exceptions. First, many extracellular proteins thought to be lost in breast cancers may actually be supplied in situ by the stroma. Second, many genes associated with proliferation and metabolism are highly expressed in culture. Hence, whereas certain aspects of breast cancer biology can be studied using breast cancer cell lines alone, others (in particular those involving factors in the extracellular space) should include additional relevant cell types.

This study revealed that, in general, basal/ER− cell lines were more representative of their respective tumours than luminal/ER+ cell lines. In addition, 60 % of cell lines in this study were ER−, as compared with only 23 % of the primary tumours (*p* < 0.0001 by two-tailed Fisher’s exact test), an overrepresentation of the ER− status in cell lines, which has been observed previously [1]. The reason for this discrepancy remains unknown. However, it may be due to the fact that most cell lines were obtained from metastatic tumours and pleural effusions and thus represent the most aggressive variants that could be adapted to culture (a trend previously reported in renal cancer [36]). We would expect this phenomenon to be especially pronounced for the ER+/luminal subtype, which is characteristically a less aggressive subtype of breast cancer. Additionally, as cells are grown in culture, the epithelial phenotype is lost in favour of more mesenchymal traits, a type of in vitro epithelial–mesenchymal transition which would result in greater transcriptional distance between the more epithelial ER+/luminal cell lines and the respective tumours [1].

Despite the transcriptional differences between cell lines and tumours, we were nonetheless interested in determining the most transcriptionally representative breast cancer cell lines. In our analysis, we found that the correlation coefficients of individual breast cancer cell lines versus tumours varied from 0.41 to 0.58. This was remarkably similar to the range of 0.43–0.60 that was observed in an analysis of ovarian cell lines and tumours [11]. Interestingly, the top correlation of any individual cell line could be exceeded by a fictional cell line composed of the averages of all cell line gene expression values (luminal, 0.52 for BT483 vs. 0.62; basal, 0.58 for HCC70 vs. 0.60) (data not shown). This points to the importance of including multiple cell lines in any analysis to ensure that any observed phenomenon is not a product of a single outlier.

A fundamental limitation of cell culture models is that the environment created by culture conditions is markedly different from the breast cancer microenvironment [9]. The loss of stromal and immune cells in culture is one major drawback of monoculture models. Emerging evidence supports the notion that tumour stromal cells play exceptionally important roles in tumour initiation, progression and metastasis [37–39]. In fact, studies have shown that depletion of fibroblast activation protein–expressing stromal cells leads to suppression of primary tumour growth and metastasis [40]. Our research indicates that loss of the stromal and immune components is the principal transcriptional difference between cell lines and tumours. It also suggests that the stroma has a unique and significant role that often is not accounted for in in vitro studies. For example, several studies have looked at the expression levels and functional roles of various Wnt antagonists (e.g., secreted frizzled-related proteins [*SFRP*s]) in cell culture, and researchers have drawn conclusions about their absence and mechanisms of action in this context [41–44]. However, given that we found the expression levels of various *SFRP*s to be high in tumours and strongly correlated with stromal scores, we should recognize that looking at these proteins in tumour cell monoculture may not be appropriate. In fact, given their roles as matricellular proteins, it would not be surprising if their effects in vivo are quite different than those observed in vitro.

In broader investigations using gene set enrichment analysis, we observed an enrichment in cell line proliferative and metabolic gene sets, similar to those reported in other studies [45–47]. The upregulation of these gene sets could be due to two phenomena: (1) malignant cellular adaptation/selection or (2) genes more highly expressed in the malignant cells are upregulated in cell lines as a result of the enrichment of this cell subtype in culture. If the latter is true, we would expect a negative correlation with stromal/tumour purity score. For one of the gene sets, DNA replication, we observed such a negative correlation with stromal score (*r* = −0.27). Thus, the expansion of malignant cells in cell culture likely plays a role in the upregulation of this gene set. However, none of the other upregulated proliferative/metabolic gene sets display this correlation. This suggests, on the one hand, that either the derivation process or the continuous culturing of cell lines selects for a highly proliferative subset of cells. On the other hand, many of the underrepresented gene sets were matrix- or immune-related and tightly correlated with stromal or immune scores, once again indicating that loss of the stromal and immune compartments has pronounced consequences in transcriptional programs observed in cell culture.

## Conclusions

Important efforts are being made to systematically compare tumours and cell lines using DNA mutation, copy number and gene expression data from a diverse spectrum of tumour types [5, 9–11, 13, 35]. In this study, we focused on breast cancer expression data and sought to identify major transcriptional differences between cell lines and tumours while accounting for variation resulting from stromal and immune components. We determined that basal cell lines are transcriptionally better models of their respective tumours than luminal cell lines. We ranked cell lines based on their transcriptional similarity to tumour samples and recommend that cell line choices be informed by this summary. We have also pointed out situations where cell line monoculture may not be the best tumour model. Fortunately, there exist many other tumour models (e.g., patient-derived xenografts, co-cultures and three-dimensional systems) that may more appropriately represent these situations. Knowing in which contexts cell lines have high or low fidelity to tumours can help direct tumour model choice, optimizing the clinical relevance of future research efforts.

## Additional files

Additional file 1: Figure S1. Unsupervised hierarchal clustering of 50 breast cancer cell lines and 1025 TCGA breast cancer tumour samples shows that cell lines cluster apart from tumour samples. Hierarchical clustering on the 5000 most variable genes was performed using 1 − *c* (where *c* is Pearson’s correlation coefficient) as the distance and Ward’s agglomeration method. Though cell lines cluster apart from tumours, basal cell lines cluster closer to their respective tumours than luminal cell lines do. Cell line clustering followed previously observed subtype divisions. (PDF 175 kb) Additional file 2:
**Top 1 % of differentially expressed genes between tumours and cell lines (with adjusted** ***p*** **values by** ***t*** **test).** (XLSX 26 kb) Additional file 3:
**Top 1 % of differentially expressed genes between tumours and cell lines after filtering out genes correlated with stromal and immune scores in tumours (absolute tumour stromal and immune scores, Pearson correlation coefficient <0.2, adjusted** ***p*** **values by** ***t*** **test).** (XLSX 29 kb) Additional file 4:
**Comparison of the frequency of select mutational events in breast cancer tumours versus cell lines.** Differences in mutational frequencies of selected genes in TCGA BRCA tumours (*n* = 503) and CCLE breast cancer cell lines (*n* = 31). (XLSX 11 kb)

## Abbreviations

CCLE: Cancer Cell Line Encyclopaedia
ER: Oestrogen receptor
ESTIMATE: Estimation of STromal and Immune cells in MAlignant Tumours using Expression data
GAGE: Generally applicable gene set enrichment
GEO: Gene Expression Omnibus
KEGG: Kyoto Encyclopedia of Genes and Genomes
PC1: Principal component 1
PC2: Principal component 2
RNA-seq: RNA sequencing
SFRP: Secreted frizzled-related protein
TCGA: The Cancer Genome Atlas
TPM: transcripts per million
