# Supplementary figures and images for: Assessing breast cancer cell lines as tumour models by comparison of mRNA expression profiles
Source: Breast Cancer Res. 2015 Aug 20;17(1):114. doi: 10.1186/s13058-015-0613-0 (PMC4545915; doi:10.1186/s13058-015-0613-0)

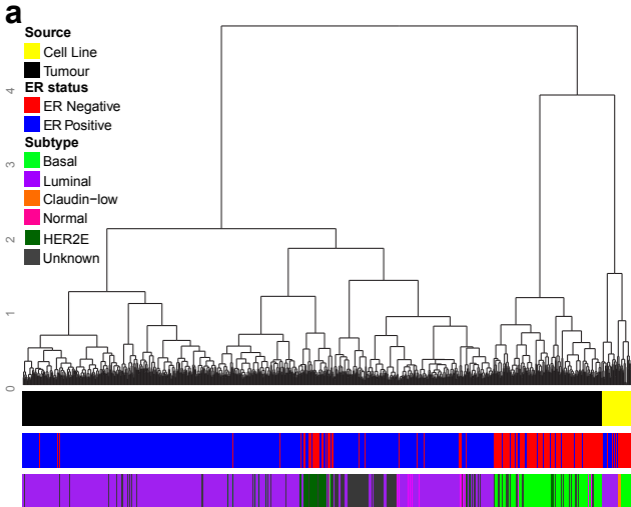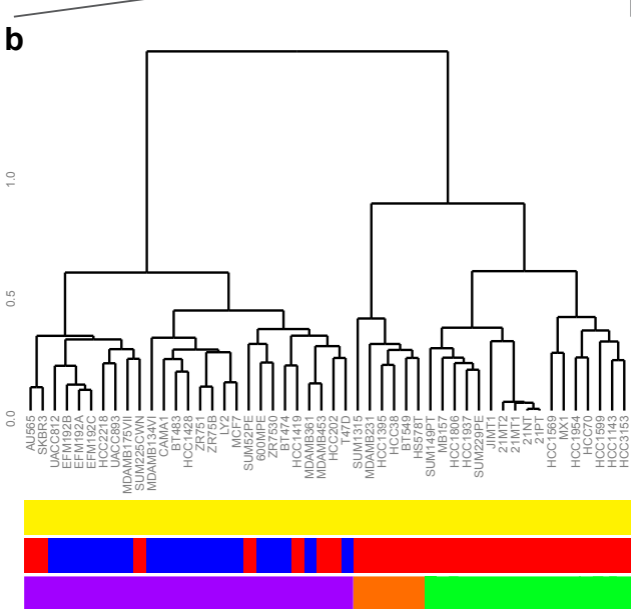

Supplement: Additional file 1: Figure S1. — Unsupervised hierarchal clustering of 50 breast cancer cell lines and 1025 TCGA breast cancer tumour samples shows that cell lines cluster apart from tumour samples. Hierarchical clustering on the 5000 most variable genes was performed using 1 − c (where c is Pearson’s correlation coefficient) as the distance and Ward’s agglomeration method. Though cell lines cluster apart from tumours, basal cell lines cluster closer to their respective tumours than luminal cell lines do. Cell line clustering followed previously observed subtype divisions. (PDF 175 kb) [file 13058_2015_613_MOESM1_ESM.pdf]
